# Laparoscopic sigmoid colectomy for transverse colonic varices due to an inferior mesenteric arteriovenous fistula

**DOI:** 10.1186/s40792-024-01911-z

**Published:** 2024-05-03

**Authors:** Taro Munechika, Keiichi Shiokawa, Issei Takeshita, Hisaaki Shimokobe, Kurumi Sahara, Yoshiko Matsumoto, Naoya Aisu, Gumpei Yoshimatsu, Suguru Hasegawa

**Affiliations:** Department of Gastroenterological Surgery, Fukuoka University Hospital, 7-45-1 Nanakuma, Jonan-Ku, Fukuoka City, Fukuoka 814-0180 Japan

**Keywords:** Inferior mesenteric arteriovenous fistula, Enteric arteriovenous fistula, Colonic varices, Case report, Surgical resection, Arteriovenous malformation

## Abstract

**Background:**

Colonic varices are a rare gastrointestinal anomaly often associated with portal hypertension. Arteriovenous fistula (AVF) in the inferior mesenteric artery (IMA) region is even rarer. Diagnosis and treatment of these entities present unique challenges, especially when the IMA is involved.

**Case presentation:**

A 48-year-old man with a history of cholecystectomy presented with after a positive fecal occult blood test. Investigations revealed varices from the splenic flexure to the transverse colon and suspected AVF in the IMA region. Given the high risk and low efficacy of endoscopic and radiological interventions, laparoscopic sigmoidectomy was performed. This surgical approach successfully addressed both the AVF and the associated varices.

**Conclusion:**

This case underscores the importance of surgical intervention for AVF and colonic varices in the IMA region, particularly when other treatment options pose high risks and have limited efficacy. The favorable postoperative outcome in this case highlights the effectiveness of carefully chosen surgical methods when managing such complex and rare conditions.

## Introduction

Colonic varices are a relatively rare condition, with only a few reported cases among gastrointestinal varices [1]. These varices are typically found in patients with portal hypertension, and they have also been reported in patients who have undergone treatment for esophageal varices and in patients who have undergone gastrointestinal surgery [2, 3].

An enteric arteriovenous fistula (AVF) is an abnormal communication between an artery and a vein within the mesentery, leading to changes in intestinal blood flow and causing complications, including gastrointestinal bleeding, abdominal pain, and ischemic enteritis.

Whereas reports of enteric AVF in the small intestine and the superior mesenteric artery region are relatively common, reports in the inferior mesenteric artery (IMA) region are rare [4]. AVF can arise from congenital factors or develop secondary to trauma or surgery. Distinguishing between congenital and acquired AVF can be particularly challenging when the condition has been present for an extended period [5, 6].

Here, we present a case of AVF in the IMA region that was associated with colonic varices and improved following surgery.

## Case presentation

A 48-year-old man was referred to our department following a positive fecal occult blood test. He had a past surgical history of cholecystectomy for cholecystitis at around 30 years of age. He had no past history of hypertension, diabetes, or liver disease. He had no history of trauma. He had a height of 163 cm and a weight of 70 kg. His abdomen was soft, flat, and non-tender. Laboratory investigations showed a white blood cell count of 5400/μL, a C-reactive protein level of 0.16 mg/dL, and a hemoglobin of 15.1 g/dL with no notable findings.

**Fig. 1:**
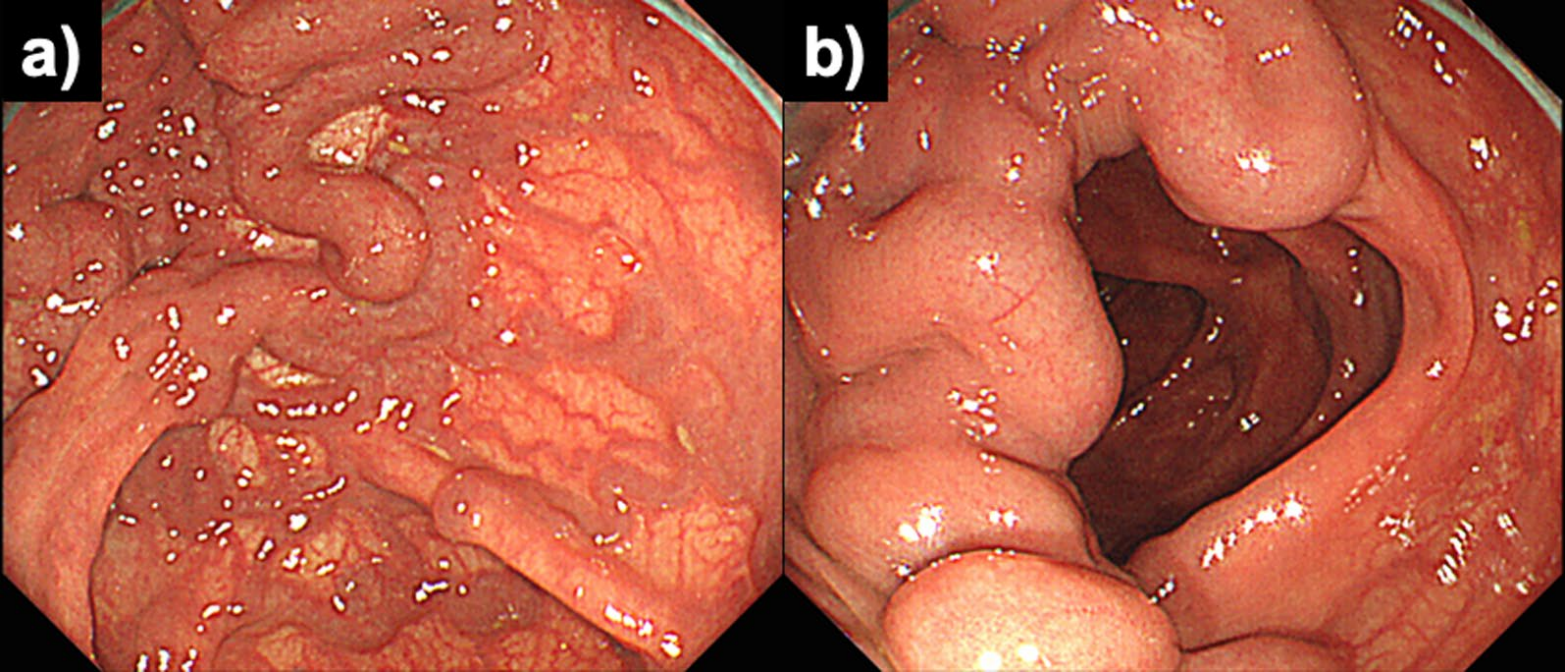
Total colonoscopy was performed. **a** Varicose veins with meandering and dilatation in the submucosal layer of the splenic flexure on the transverse colon were identified. **b** Varicose veins had no active bleeding and no red color sign

**Fig. 2:**
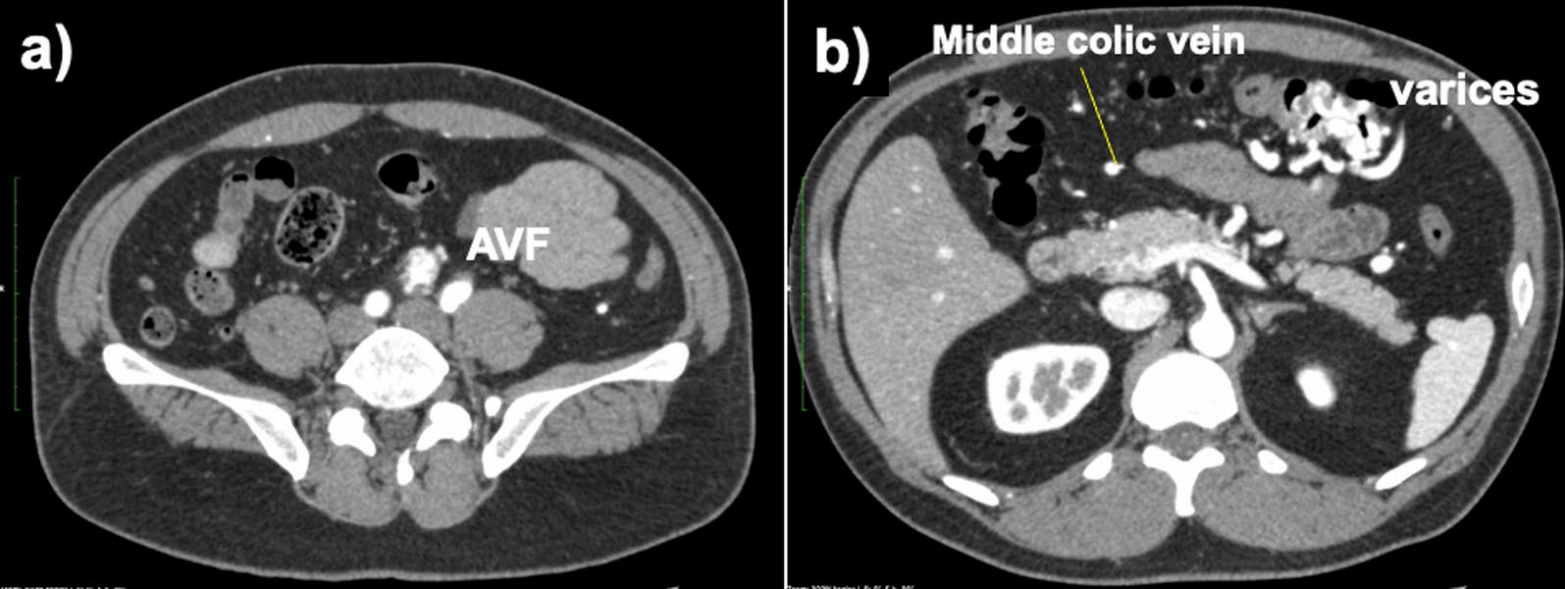
Contrast-enhanced CT was performed. **a** Early enhancement of the inferior mesenteric vein was observed as well as inferior mesenteric artery in the arterial phase. **b** Varicose veins were observed in the splenic flexure, communicating with the inferior mesenteric vein and the middle colic vein

He underwent colonoscopy, which revealed varices from the middle of the transverse colon to the splenic flexure. There was no active bleeding or red color sign in the varices (Fig. 1). A contrast-enhanced computed tomography (CT) examination revealed early intense contrast enhancement of the inferior mesenteric vein (IMV) as well as IMA and abnormally dilated and tortuous vessels around the left side of the transverse colon with early intense staining in the arterial phase (Fig. 2).

**Fig. 3:**
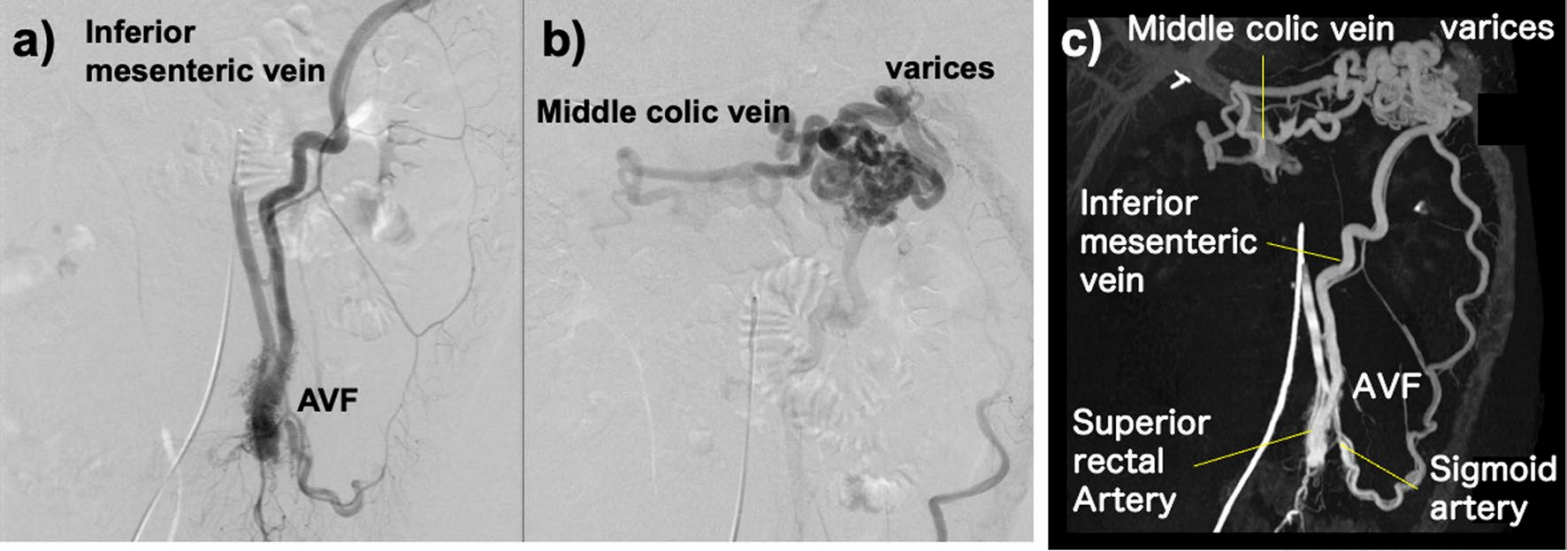
Selective digital subtraction angiography was performed to confirm the specific hemodynamics on the patient. **a** Selective inferior mesenteric arterial angiography revealed an arteriovenous fistula between inferior mesenteric artery and vein. **b** Inferior mesenteric vein flew into the middle colic vein through the transverse colonic varices at the splenic flexure. **c** Angioarchitectonic images showed an arteriovenous fistula forming between the superior rectal artery and the sigmoid colon artery and inferior mesenteric vein. The marginal veins of the descending colon were dilated as well as inferior mesenteric vein

Colonic varices, therefore, were suspected because of the abnormal blood flow in the IMA. And angiography was performed to investigate it further. Selective digital subtraction angiography of the IMA revealed an AVF between the first branch of the sigmoid artery and the IMV. Contrast flowed into the IMV immediately after the IMA through the first branch of the sigmoid artery and the IMV became dilated. And blood in the IMV flowed into the mesocolic vein around the splenic flexure, and tortuous and dilated veins, which were considered to be varicosities, were found around the left side of the transverse colon. Blood in the IMV then flowed into the portal vein via the colonic varices but not into the splenic vein directly (Fig. 3).

Finally, we diagnosed colonic varices caused by AVF between the first artery in the sigmoid colon and the IMV as the reason for the positive fecal occult blood test. Endoscopic treatment targeting only varicosities while leaving an AVF intact is associated with a high risk of perforation and recurrence. Radiological intervention was considered, but the large diameter of the vessel posed challenges for embolization, further increasing the likelihood of recurrence. Furthermore, the proximity of the AVF to the intestinal tract raised concerns about potential obstruction of blood flow following embolization. Therefore, the decision was made to perform laparoscopic sigmoidectomy.

**Fig. 4:**
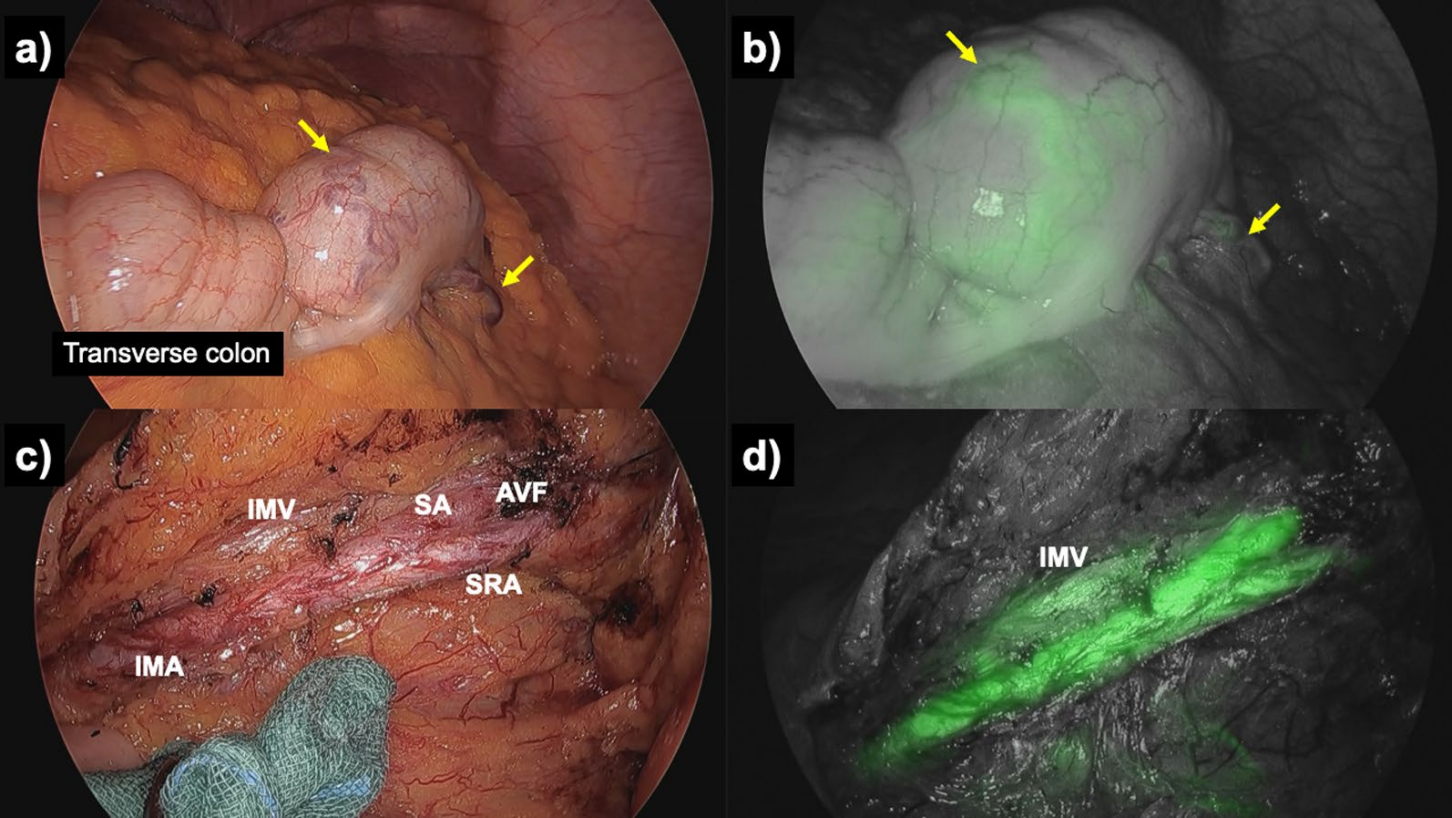
Intraoperative findings before resection of the arteriovenous fistula were demonstrated. **a**, **b** Veins and varicosities (yellow arrows) on the wall of the transverse colon were laparoscopically demonstrated. The indocyanine green fluorescence on the dilated veins and varicosities had been seen through the inferior mesenteric vein immediately after observation of the inferior mesenteric artery. **c**, **d** Blood vessels around the inferior mesenteric artery and vein were exposed to determine the specific hemodynamics. Indocyanine green fluorescence on the inferior mesenteric vein was observed immediately after inferior mesenteric artery in advance of the marginal veins of the sigmoid colon

**Fig. 5:**
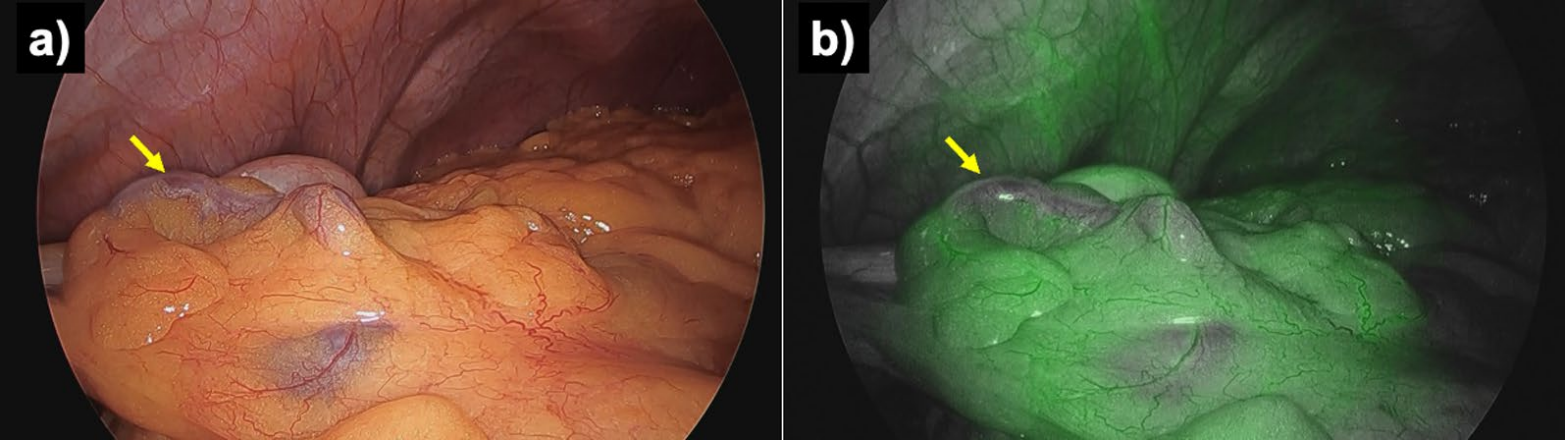
Intraoperative findings after excision of the arteriovenous fistula with sigmoid colon. **a**, **b** Indocyanine green fluorescence did not immediately flow into varices of the transverse colon (yellow arrows) after recognition of the inferior mesenteric artery

The surgery was performed under general anesthesia in the lithotomy position. Preoperative CT showed that the AVF was located at the bifurcation of the superior rectal artery (SRA) and sigmoid artery. The left side of the colonic mesentery, including the pedicle of the IMA and IMV, was mobilized by a medial approach. The IMA, SRA, first branch of the sigmoid artery, and IMV were exposed and separated from the mesenteric adipose tissue. Blood flow was evaluated by intravenous injection of fluorescent indocyanine green (ICG) dye. ICG fluorescence in the IMV was seen immediately after observation of the IMA and SRA under near-infrared spectroscopy. Tortuous and dilated colonic varices were also visualized on the left side of the transverse colon after observation of ICG fluorescence in the IMV. Considering that the AVF did not involve the IMA or left colic artery, the SRA and first branch of the sigmoid artery were dissected with preservation of the left colic artery (Fig. 4). The sigmoid colon and mesentery were resected, and a colorectal anastomosis was created using the double stapling technique. After reconstruction, return of blood flow was confirmed by ICG fluorescence, and early fluorescence of varicosities in the transverse colon was no longer observed (Fig. 5).

**Fig. 6:**
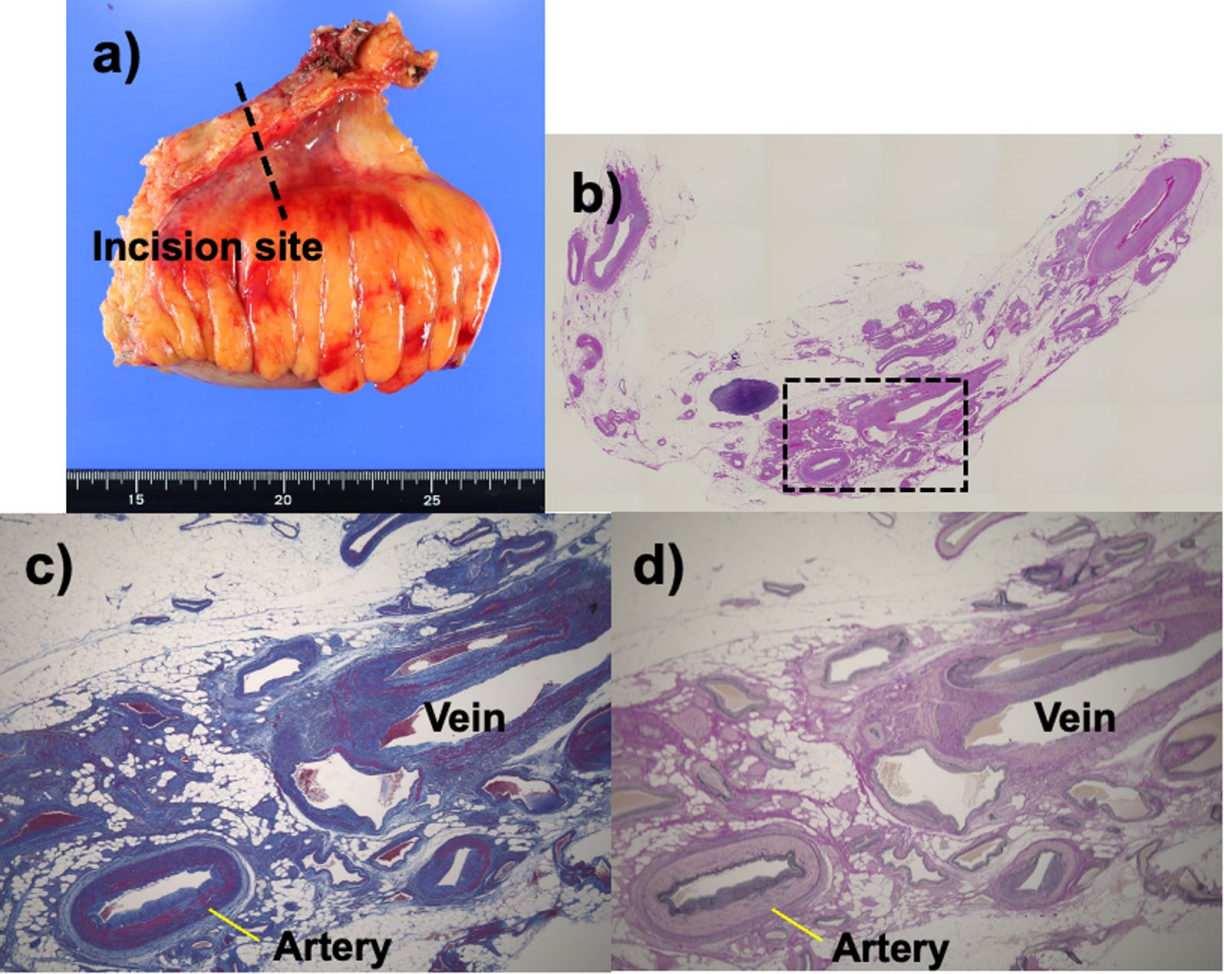
Pathological findings of the resected specimen. **a** No obvious arteriovenous fistula was macroscopically identified in the resected specimen. **b** In the hematoxylin and eosin staining, large and small vessels were complicatedly existed. **c**, **d** The wall of the vein was irregularly thickened, and Masson’s Trichrome staining and Elastica van Gieson staining could not reveal the definitive evidence of the arteriovenous fistula

**Fig. 7:**
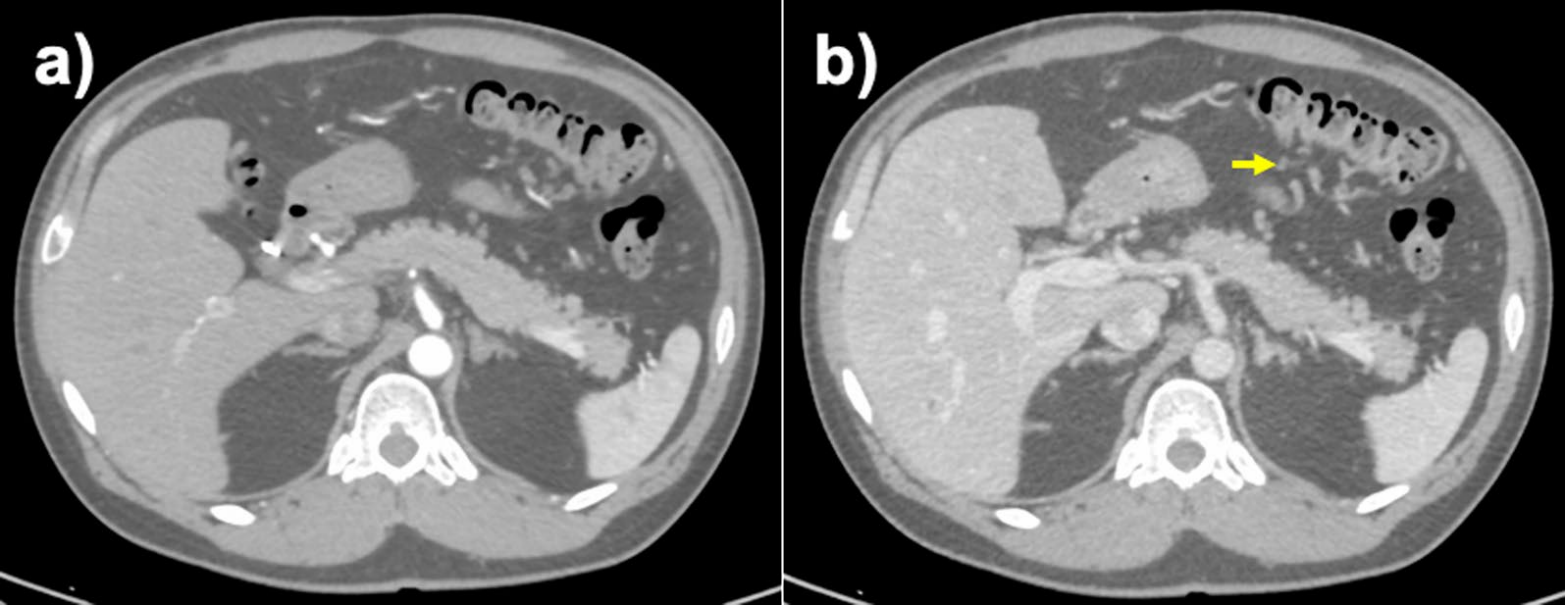
Contrast-enhanced CT examination was performed at 3 months after surgery. **a** In the arterial phase, transverse colonic varices (yellow arrows) were not enhanced. **b** In the portal venous phase, the transverse colonic varices were gradually enhanced

**Fig. 8:**
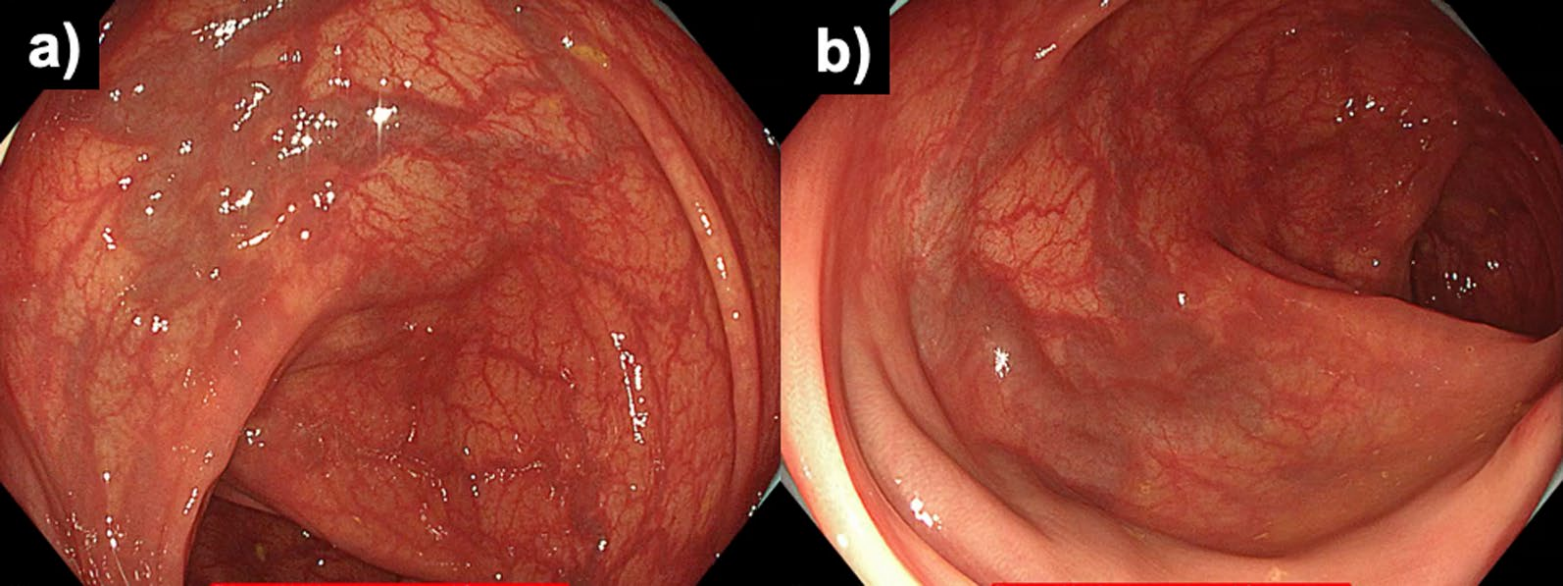
Colonoscopy was performed at 6 months after surgery. **a**, **b** At the splenic flexure of the transverse colon, vascular dilatation on the varices was improved

In the resected specimen, no obvious AVF was macroscopically identified, however irregularly thickened veins on the mesentery were microscopically observed around the IMA in the hematoxylin and eosin (HE) stain and large and small blood vessels were complicatedly existed. Some veins contained the organized-thrombus. The Masson's trichrome stain and Elastica van Gieson stain could not identified the fusion vessel as the definitive evidence of the AVF, however the characteristics of the vessels suggested the presence of AVF or AVM (Fig. 6). The postoperative clinical course was favorable, and he was discharged on postoperative day 10. Contrast-enhanced CT at 3 months after surgery showed that early enhancement of IMV was no longer observed in the arterial phase, and that transverse colonic varices were not enhanced in the arterial phase. The dilatation of the transverse colonic varices were obviously improved, although the colonic varices were morphologically remained (Fig. 7). Colonoscopy at 6 months after surgery confirmed improvement of the varices by resection of AVF with sigmoidectomy (Fig. 8).

## Discussion

Leaving varices untreated may lead to significant bleeding, so intervention is often necessary. Esophageal varices associated with portal hypertension resulting from cirrhosis are common. In those cases, local endoscopic treatment such as endoscopic variceal ligation therapy (EVL) and endoscopic variceal sclerotherapy (EIS) are performed. Radiological interventions (IVR), such as balloon-occluded retrograde transvenous obliteration aimed at addressing the underlying cause of increased venous pressure may also be performed to prevent variceal bleeding. When treating colonic varices, it should be borne in mind that the risk of severe complications, particularly perforation, is much higher in the lower gastrointestinal tract than in the upper gastrointestinal tract. Moreover, the risk of recurrence would be high if the local endoscopic treatment, such as EVL or EIS, is performed with ignoring the underlying cause of the varices. In our case, elevated venous pressure in the IMV caused by an AVF in the IMA region was the underlying factor. In this present case, the cause of the transverse colonic varices was the elevated venous blood pressure due to the AVF. Therefore, we considered that excising the AVF could lead to an improvement in the varices. Regarding the other treatment, IVR may be one of the options in the point of view of invasiveness compared to surgical treatment, however coil embolism can cause post-procedural complications, including intra-abdominal bleeding [7] and intestinal ischemia [4], emphasizing the need to consider changes in blood flow after treatment. Moreover, in this case, the diameter of the fistula was relatively large and the distal portion of the AVF was in close proximity to the colon, so blood flow could impair if an embolus flowed into peripheral blood vessels. Regarding surgical treatment, ligating or resecting the AVF alone using surgical clips was also difficult because the distal side of the AVF was close to the marginal blood vessels of the sigmoid colon and was likely to cause ischemic complications on the colon.

AVF in the IMA region is a rare entity, and its causes have often been unclear in the small number of cases reported. Acquired factors, including trauma, postoperative complications, and cancer, are cited, with postoperative iatrogenic causes being more prevalent. Congenital cases are known as colonic arteriovenous malformations, where an arteriovenous shunt forms in an abnormal vascular network called a nidus. Although a nidus was not pathologically identified, the absence of a history of intestinal surgery or trauma in the present case made it difficult to determine whether the condition was derived from the congenital or acquired reason.

In general, IMV typically drains into the splenic vein or the portal vein. Although there are some reports of a collateral circulation developing between the IMV and the middle colic vein in the context of portal hypertension, however, in our case, the radiological examination did not reveal the direct flow of the contrast from the IMV to the portal or splenic vein. Therefore, it is presumed that the IMV originally drained into the middle colic vein. This anatomical feature with a specific hemodynamics likely led to the formation of varices in the splenic flexure area.

Whether to excise the area of the intestine with varices is a matter of debate. In this case, the variceal region on the colon was not excised because the goal was to decrease venous pressure after excision of the AVF. Use of ICG fluorescence imaging was important in this case for real-time visualization of vascular dynamics during surgery. The usefulness of ICG during resection of arteriovenous malformations has been reported [8]. ICG imaging confirmed successful exclusion of the AVF and subsequent reduction in venous pressure, providing direct evidence of the efficacy of surgical intervention. This technique not only enhanced the precision of the procedure but also potentially reduced the risk of postoperative complications by immediate identification of vascular abnormalities. Postoperative imaging analysis and endoscopic observation confirmed disappearance of the varices, suggesting the effectiveness of the reduction in venous pressure achieved by resection of sigmoid colon and mesentery with AVF.

A search of the PubMed database from 2013 to 2023 using the key terms “arteriovenous fistula”, “colon”, and “arteriovenous malformation” yielded 25 case reports on inferior mesenteric AVF. We briefly reviewed these cases, with inclusion of our present case (Table 1). While reports exist of cases presenting with AVF and varices, in those instances both were present in the cecum and were excised together [9]. Our present case is the only instance in which a colonic varix was treated by excising an AVF located at another site. The median patient age was relatively young at 56 years (range 24–81). Twenty-one of the 26 cases were in men, indicating a male predominance. The most common primary symptoms were abdominal pain and diarrhea, with many cases suspected to be ischemic colitis. A presumed cause was recorded in eight cases, four of which were post-colectomy for colon cancer and one post-hysterectomy, suggesting a significant proportion of postoperative cases. Treatment involved bowel resection in 14 cases, colostomy in one, and embolization in ten. In a case, surgery was scheduled after embolization [10]. A further case was treated conservatively [5]. Five of 9 patients who underwent embolization experienced recurrence of symptoms, ischemia, or rupture of varices, leading to subsequent surgical intervention [4, 7, 11–13]. There have been reports of embolization material deviating peripherally in AVFs with a diameter of ≥ 8 mm [4], suggesting that even when embolization is considered, surgery may ultimately be necessary.

**Table 1:** Literature review of the inferior mesenteric arteriovenous fistula

	Authors (year)	Age	Sex	Chief complaint	Underlying cause of AVF	AVF				Varices		
						Location of AVF	Solitary/multiple	Treatment	Complications	Presence of varices	Location of varices	Treatment of varices
1	Somasundaram et al. (2013)	60	F	Fresh blood per rectum	Hysterectomy	SRA	Solitary	HAR	–	◯	RS	Resection
2	Athanasiou et al. (2014)	66	M	Intestinal obstruction	ー	IMA-SRA	Multiple	Embolization → LHC	Atrophy and ischemia	×	–	–
3	Ushigome et al. (2014)	60	M	Diarrhea	Sigmoidectomy	SRA	Solitary	HAR	–	◯	RS	Resection
4	Langroudi et al. (2015)	48	F	Abdominal pain	ー	IMA	Solitary	Surgical resection	–	◯	Mesentery(IMA)	Resection
5	Coulier et al. (2016)	45	M	Abdominal pain	ー	LCA-SRA	Multiple	Embolization → LHC	Recurrence of symptoms	◯	D-S	Resection
6	Kamo et al. (2016)	51	M	Abdominal pain and melena	ー	LCA	Solitary	Embolization	–	◯	T-D	Embolization
7	Cheng et al. (2017)	62	M	Abdominal discomfort	ー	IMA	Solitary	LHC	–	◯	D-RS	Resection
8	Strjina and Kelley (2017)	48	M	Abdominal pain and diarrhea	ー	IMA	Solitary	LAR	–	◯	S	Resection
9	Gelonch et al. (2017)	73	M	Abdominal pain and diarrhea	ー	SA	Multiple	Embolization → LHC (planned)	–	◯	D-S	Embolization
10	Lee et al. (2017)	56	M	Abdominal pain and diarrhea	ー	IMA	Solitary	LHC	–	◯	T-D	Unclear
11	Matsuda et al. (2020)	65	M	Abdominal pain	Mesenteric inflammatory veno-occlusive disease	LCA	Solitary	LHC	–	◯	D-S	Resection
12	Das Gupta et al. (2019)	46	M	Abdominal pain	Severe portal hypertension and cardiomyopathy	SRA	Solitary	Embolization × 2 → Sigmoidectomy	Recurrence of symptoms	×	–	–
13	Doi et al. (2020)	81	M	Diarrhea	Left hemicolectomy + bevacizumab	IMA	Solitary	Embolization	–	×	–	–
14	Charalambous et al. (2020)	73	F	Diarrhea and abdominal pain	Right hemicolectomy	IMA	Solitary	Embolization × 3	–	◯	T-D	Embolization
15	Cubisino et al. (2021)	72	M	Diarrhea and abdominal pain	ー	LCA	Solitary	Observation	–	◯	T	Observation
16	Shah et al. (2021)	24	F	Colitis	Idiopathic myointimal hyperplasia	IMA-SRA	Multiple	Embolization → LHC	Recurrence of symptoms	×	–	–
17	Kimura et al. (2021)	50	M	Abdominal pain and melena	ー	SRA	Solitary	LAR	–	◯	D-RS	Unclear
18	Hamaguchi et al. (2022)	50	M	Abdominal pain	Sigmoidectomy	SRA	Multiple	Colostomy	–	×	–	–
19	Hirota et al. (2022)	64	M	Fever	ー	IMA	Solitary	Embolization → surgical suture	Rupture	◯	S-RS	Embolization
20	Shiraishi et al. (2022)	70	M	Abdominal pain and melena	ー	SA-SRA	Multiple	LAR	–	◯	D-RS	Resection
21	Maqboul et al. (2023)	40	M	Abdominal pain and diarrhea	clostridium difficile colitis and intestinal amebiasis	IMA	Multiple	LHC	Bleeding	×	–	–
22	Chang et al. (2023)	67	M	Abdominal pain	ー	IMA	Solitary	LHC	–	◯	T-D	Resection
23	Njoum et al. (2023)	56	M	Bleeding	ー	LCA	Multiple	LHC	–	◯	D	Resection
24	Trieu et al. (2023)	32	F	Bleeding	ー	SA-SRA	Multiple	LAR	–	Unclear	–	–
25	Saito et al. (2023)	50	M	Abdominal distension	ー	IMA	Multiple	Embolization	–	◯	T-S	Embolization
26	Our case	48	M	Fecal occult blood positive	ー	IMA-SRA	Solitary	Sigmoidectomy	–	◯	T-D	None

SRA: superior rectal artery; IMA inferior mesenteric artery; LCA: left colic artery; SA: sigmoid colon artery; HAR: high anterior resection; LHC: left hemicolectomy; LAR: low anterior resection; D: descending colon; S: sigmoid colon; T: transverse colon; RS: rectosigmoid

The uniqueness of this case lies in the fact that the AVF in the IMA region was the cause, and sigmoidectomy was performed to treat transverse colonic varices with preserving the transverse colon. Furthermore, changes in blood flow were confirmed intraoperatively using ICG fluorescence imaging. The postoperative course also allowed observation of the healing process of the varices over time, making this a rare report.

## Data Availability

Not applicable.

## Abbreviations

AVF: Arteriovenous fistula
IMA: Inferior mesenteric artery
IMV: Inferior mesenteric vein
SRA: Superior rectal artery
LCA: Left colic artery
ICG: Indocyanine green
